# No-Tillage System Can Improve Soybean Grain Production More Than Conventional Tillage System

**DOI:** 10.3390/plants12213762

**Published:** 2023-11-03

**Authors:** Gustavo Ferreira da Silva, Juliano Carlos Calonego, Bruno Cesar Ottoboni Luperini, Vinicius Brasil Silveira, Larissa Chamma, Rogério Peres Soratto, Fernando Ferrari Putti

**Affiliations:** 1 Department of Crop Science, College of Agricultural Sciences, São Paulo State University (UNESP), Botucatu 18610-034, Brazil; juliano.calonego@unesp.br (J.C.C.); vinicius_brs@hotmail.com (V.B.S.); larissa.chamma@hotmail.com (L.C.); rogerio.soratto@unesp.br (R.P.S.); 2 Department of Agricultural Engineering, College of Agricultural Sciences, São Paulo State University (UNESP), Botucatu 18610-034, Brazil; b.luperini@unesp.br (B.C.O.L.); fernando.putti@unesp.br (F.F.P.); 3 Department of Biosystems Engineering, School of Sciences and Engineering, São Paulo State University (UNESP), Tupã 17602-496, Brazil

**Keywords:** conservationist management, *Glycine max*, no-tillage, soil physics, tillage, water stress

## Abstract

Soil management systems can directly interfere with crop yield via changes in the soil’s physical and hydraulic properties. However, short- to medium-term experiments of conduction do not always demonstrate the modifications of the management systems in these properties. Thus, the aim of this study was to evaluate the physical properties of the soil in a long-term management system and to relate it to the storage and availability of water to plants, verifying its effect on soybean yield. The experiment was conducted in randomized blocks in a split-plot scheme with four replications. Plots were composed by soil management (conventional tillage and no-tillage), and subplots represented three soil depths (0.0–0.1, 0.1–0.2, and 0.2–0.4 m). The soil’s physical and hydraulic properties, root development, and soybean yield were evaluated. The no-tillage system not only presented higher bulk density and soil resistance to compaction up to a depth of 0.2 m but also greater root development. This management also did not affect the process of water infiltration in the soil and presented an increase in soybean grain yield by 6.5%. The long-term no-tillage system (33 years) offers less risk of water stress to soybean plants; it contributes to greater grain yield of this crop when compared to the conventional tillage system.

## 1. Introduction

The improvement and maintenance of the physical quality of the soil must be a fundamental requirement in the management systems of agricultural areas, as it is a determining factor in crop productivity directly linked to the soil storage capacity and availability of water to plants [1,2].

Several studies have reported the effects of conventional tillage (CT) and no-tillage (NT) systems on soil physical properties but without relating them to the availability of water to plants during crop cultivation [3,4,5]. Awal et al. [3] demonstrated that there is spatial variability in the physical properties of soil in long-term no-tillage, but they did not study the effects of these variations on crop performance. Galdos et al. [4] reported that soils under long-term zero-tillage present better pore connectivity and higher total porosity than conventional tillage; however, this work did not demonstrate the impacts of these improvements on the development and productivity of cultivated plants. Mondal and Chakraborty, via research with meta-analysis, confirmed that no-tillage practices contribute to soil structural development and an increase in water retention pores, even over a short-term period, however, they did not report the effects of these soil changes on the development of agricultural crops. Usually, in CT areas, the soil has a lower rate of water infiltration, lower water storage capacity, and lower water availability to plants, since it is a system with greater vulnerability to crops in periods of heavy rains or drought [6,7,8]. In this management system, the soil is frequently turned, modifying its physical and hydraulic properties in a short time, with the layer being frequently compacted between 0.10 and 0.20 m [9,10].

On the other hand, areas of cultivation under NT generally present the surface layers more compacted due to the traffic of machines and agricultural implements without the mechanized tillage of the soil. The higher soil bulk density in NT contributes to the reduction in total porosity due to the reduction in macropores and increase in micropores, which are mainly responsible for water retention in the soil [6,11]. However, not disturbing the soil provides a continued soil pore network, making porosity more efficient for liquid and gaseous movement [12,13]. Meanwhile, frequent turning in CT pulverizes the soil, disrupting the connectivity and structure of the pores [4,14].

In the literature, there are several research studies that evaluate the physical and hydraulic properties and the performance of agricultural crops in CT and NT; however, these studies have reported contradictory results [15,16]. Most of the time, this is related to the time of adoption of soil management, and studies conducted in long-term systems generate more reliable results [8,15,17]. Recent studies conducted in tropical soils reinforce this theory, demonstrating that long-term NT promotes improvements in the physical quality of the soil, resulting in an increase in grain productivity [18,19,20,21]. Nevertheless, there are few works carried out in areas with long-term NT, and research in these systems that relate soil physics results with crop productivity is also scarce [22,23].

In this sense, with this research, we seek to demonstrate the effects of soil management systems on the soil’s physical and hydraulic properties and relate them to root development and soybean grain yield. Therefore, the hypothesis of this study is that areas managed under long-term NT have better physical and hydraulic soil conditions, resulting in higher soybean productivity. That is because there is a greater continuity of pores in NT [4], and the presence of permanent soil cover reduces the impact of raindrops on the surface, surface runoff, and water evaporation [24]. In addition to these factors, the higher organic matter content in the surface layers contributes to higher porosity, favoring a higher rate of water infiltration into the soil [25,26].

Therefore, the objective of this study is to evaluate the physical properties of the soil in a long-term management system and to relate them to the storage and availability of water to plants, verifying its effect on the productivity of the soybean crop.

## 2. Results

### 2.1. Soil Physical and Hydraulic Properties and Root Development

The values of soil penetration resistance (PR), bulk density (Bd), maximum available water capacity (MWC), and root dry biomass (RDB) showed significant interaction between soil management systems and soil depth (Table 1).

For PR, there was a significant difference (*p* < 0.05) between the soil management systems in the 0.00–0.10 and 0.10–0.20 m layers. The PR values obtained in NT were 4.11 and 4.40 MPa in these respective layers, which were values 5.8 and 2.7 times superior to those obtained in CT. In CT, the highest value of RP occurred in the 0.20–0.40 m layer, while in NT, the highest PR was in the 0.10–0.20 m layer, however, without differing from the PR in 0.00–0.10 m.

When comparing the Bd results between management systems, there was a significant difference only in the 0.00–0.10 m layer, with Bd values of 1.04 and 1.30 g cm^−3^ in CT and NT, respectively. The Bd in CT was lower in the most superficial layer (0.00–0.10 m), while in NT, the lowest value was obtained in the 0.20–0.40 m layer, with 1.26 g cm^−3^, differing only from the NT of 1.35 g cm^−3^ obtained in the 0.10–0.20 m layer.

In CT, the highest MWC was obtained in the 0.10–0.20 m layer, with 0.069 cm^3^ cm^−3^, while the lowest MWC happened in the 0.20–0.40 m layer, with 0.047 cm^3^ cm^−3^ (Table 1). In NT, the highest MWC occurred in the 0.20–0.40 m layer, followed by the 0.00–0.10 m layer. In the comparison between the soil management systems, in the first two layers, from 0.00 to 0.20 m, the highest MWC occurred in CT. In the 0.20–0.40 m layer, the highest MWC occurred in NT.

As for RDB in CT, the largest weight occurred at a depth of 0.00 to 0.10 m, with 1110 kg ha^−1^, which represents 85.7% of the total root dry biomass in the profile evaluated in this management system (Table 1). In NT, the highest RDB occurred in the 0.00–0.10 m and 0.10–0.20 m layers, with 1316 and 1009 kg ha^−1^, that is, respectively, 53.6 and 41.1% of the total RDM produced from 0.00 to 0.40 m. In the comparison between management systems, NT differed from CT in terms of RDB only in the depth of 0.10–0.20 m, producing 7.4 times more roots than in CT.

For the values of macroporosity (Mp), microporosity (mp), and total porosity (Tp) of the soil, there was no significant interaction between the tested factors (Table 2). There was a significant difference only for Mp in the comparison between management systems, with higher Mp in CT, where this system provided Mp of 0.09 cm^3^ cm^−3^, that is, 33% higher than the Mp obtained in NT.

### 2.2. Infiltration, Storage, and Availability of Water in the Soil

**Figure 1:**
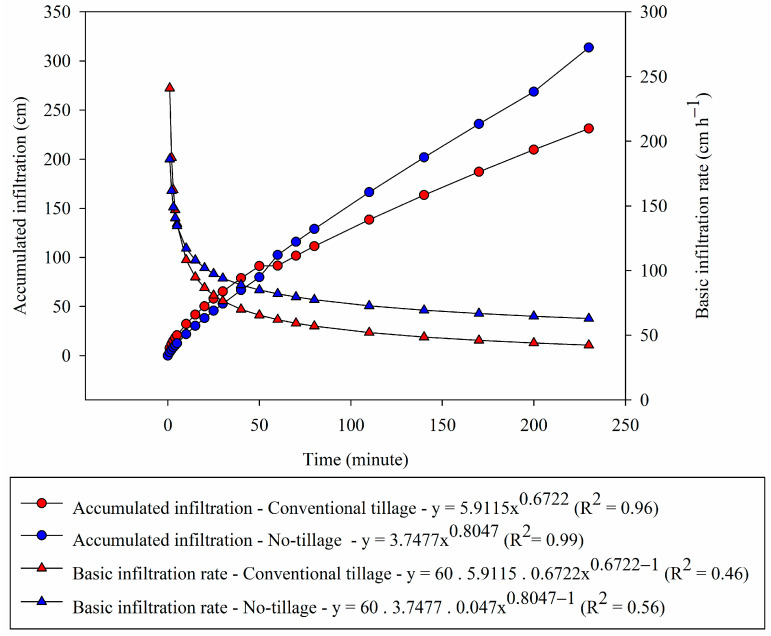
Accumulated infiltration and basic infiltration rate of water into the soil in conventional tillage and long-term no-tillage systems.

The soil water infiltration (WI) was similar between soil management systems up to 150 min (Figure 1). After this time, there was a greater WI in the NT, meaning that at the end of the evaluated period, NT showed approximately 33% more infiltrated water than CT. For the CT treatment, k (5.9116) was higher, providing a lower basic infiltration rate (BIR), as this parameter is related to the initial conditions of the soil. The smaller n related to soil characteristics causes lower BIR (Figure 1).

The WI accumulated in the first 55 min was greater in the CT (Figure 1). However, from that moment on, the increments in the WI in NT were greater than in CT. At the end of the WI test, in NT, there was a total infiltration of 314 mm of water; this value was 26% higher than the WI in CT. 

CT initially showed high BIR in the soil. In the first minute of infiltration, BIR in CT was 241.02 cm h^−1^, while in NT, it was 186.12 cm h^−1^, with BIR in CT 29.50% higher than in NT (Figure 1). However, after ten minutes of water infiltration, the NT showed a higher BIR, with a constant increase until the first 60 min, after which the values remained practically constant, 30% higher than in the CT.

**Figure 2:**
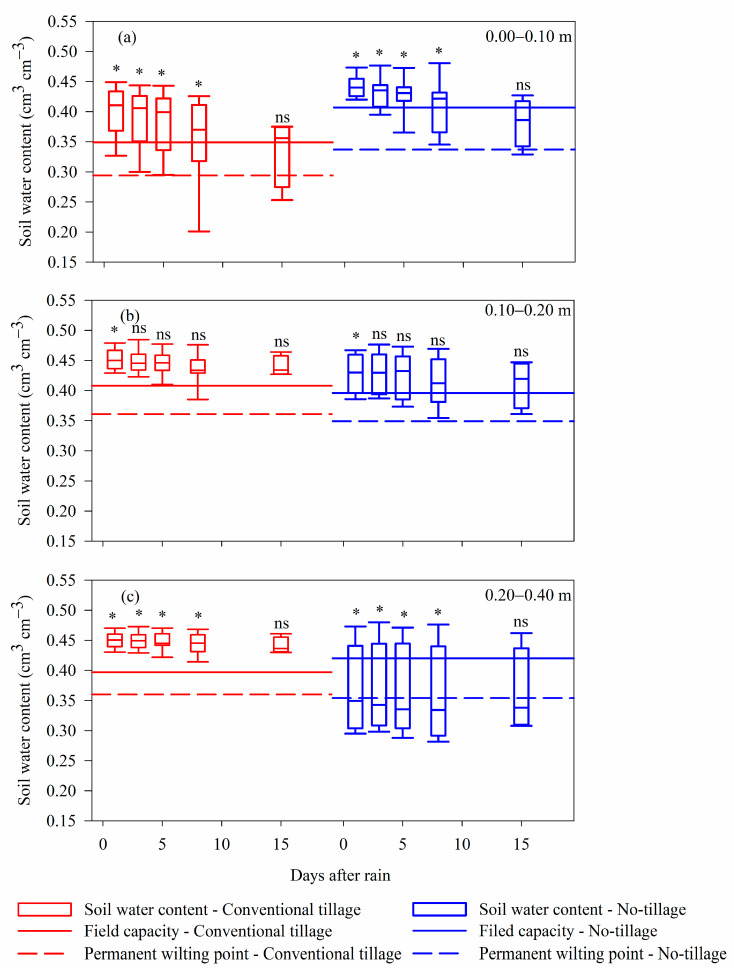
Water content stored in the soil and water available for absorption by plants (content between field capacity and permanent wilting point) in days after rainfall (DAR) in conventional tillage and long-term no-tillage systems at depths of 0.00–0.10 m (**a**), 0.10–0.20 m, (**b**) and 0.20–0.40 m (**c**). *: management systems differ from each other on the same DAR reading via the Mann–Whitney test (*p* < 0.05); ns: management systems do not differ from each other on the same DAR reading via the Mann–Whitney test (*p* < 0.05).

The soil water content (WC), at a depth of 0.00–0.10 m, differed between management systems at one, three, five, and eight days after rain (DAR), with higher WC stored in NT (Figure 2). However, WC values were above field capacity (FC) in both soil management systems. The exception was 15 DAR, where in NT, WC was within the range of water availability to plants (between FC and permanent wilting point (PWP)), but without statistical difference.

At a depth of 0.10–0.20 m, there was a difference in the WC stored only at the one DAR, in which the CT presented a higher WC (Figure 2). Yet, in both systems, the water storage was above the FC. Despite the storage capacity being the same between the management systems (0.047 cm^3^ cm^−3^), the critical value for water uptake by plants (PWP) in NT was lower (0.349 cm^3^ cm^−3^).

At the last evaluated depth (0.20–0.40 m), the NT stored water below the permanent wilting point on all days after the rain, but with no statistical difference at 15 DAR (Figure 2). Meanwhile, the CT presented WC above the FC.

**Figure 3:**
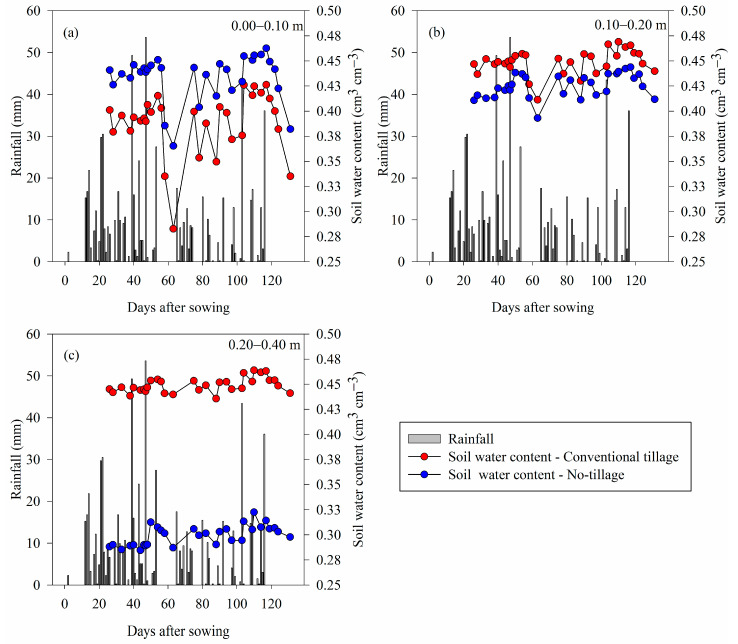
Variation in water stored in the soil and precipitation volume during the soybean cultivation cycle in conventional tillage and long-term no-tillage systems at depths of 0.00–0.10 m (**a**), 0.10–0.20 m (**b**), and 0.20–0.40 m (**c**). To compare conventional tillage and no-tillage, the Mann–Whitney test was applied (*p* < 0.01).

Regarding the WC stored in the soil during the soybean cycle, in the 0.00 to 0.10 m layer, the WC stored right after rainfall had a similar behavior between the management but with greater storage in the NT (Figure 3). Nevertheless, after the rain, the soil under CT dried faster than the soil under NT in this more superficial layer.

At depths of 0.10–0.20 and 0.20–0.40 m, the difference was significant (*p* < 0.01) (Figure 3); the WC stored over time was higher in the CT, with a greater difference at the last depth, with a volume of water stored around 33% higher than NT. However, at depths of 0.00–0.10 and 0.20–0.40 m, CT presents a higher drying speed (Figure 3).

In general, NT has higher water storage and availability in the 0.00–0.20 m layer when compared to CT (Figure 2 and Figure 3).

### 2.3. Soybean Yield

**Figure 4:**
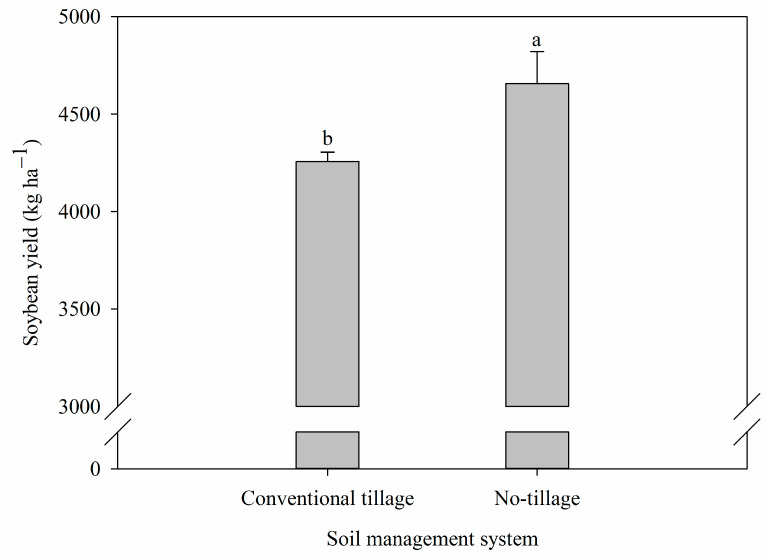
Soybean grain yield grown in a long-term experiment with conventional tillage and no-tillage systems. Different letters differ from each other by Student’s *t*-test (*p* < 0.05).

Soybean grain yield was higher in NT, with an average of 4556 kg ha^−1^, 6.5% higher than in CT (Figure 4).

### 2.4. Correlation between Soil Physical and Hydraulic Properties, Soil Water, and Soybean Yield

Soybean grain yield showed a significant and positive correlation with macroporosity and a negative correlation with microporosity at a depth of 0.10–0.20 m (Table 3).

At 0.00–0.10 m depth, it was possible to observe that the RDB had no significant correlation with any of the variables. In the depth of 0.10–0.20, there was a positive correlation with the PR and with WI (Table 3).

At a depth of 0.10–0.20 m, RDB also correlated with MWC but negatively. At a depth of 0.20–0.40 m, it showed a positive correlation with MWC and WI (Table 3).

PR correlated negatively with Tp, Mp, and MWC at 0.00–0.10 m depth (Table 3). At a depth of 0.10–0.20 m, there was also a negative correlation with the MWC, confirming the results found at the previous depth. However, at these depths, there was also a positive correlation between PR and WI.

Tp showed a significant correlation with Mp and MWC in the 0.00–0.10 m depth and with Mp and mp in the 0.20–0.40 m layer (Table 3). At a depth of 0.00–0.10 m, it was possible to observe a negative correlation between Mp and WI. There was also a negative correlation with WSS.

At a depth of 0.10–0.20 m, a negative correlation between Mp and mp was observed (Table 3). At a depth of 0.20–0.40 m, Mp showed a positive correlation only with WSS. The mp, at a depth of 0.10–0.20 m, showed a negative correlation only with the yield of soybeans.

Bd, in the initial depth (0.00–0.10), presented a negative correlation with MWC and a positive one with WI (Table 3). At depths of 0.00–0.10 and 0.10–0.20 m, MWC had a negative correlation with WI. At these same depths, there was also a negative correlation between PR and MWC. In the 0.20–0.40 cm layer, MWC was positively correlated with WI.

## 3. Discussion

### 3.1. Soil Physical and Hydraulic Properties and Soybean Performance

The variation in soil properties and soil water content between NT and CT directly influenced soybean grain productivity (Figure 4 and Table 3). The NT improved grain production more than a conventional tillage system, and the variation in soil properties and soil water content was the influential mechanism. The highest values of PR and Bd in NT were already expected, considering that in this management system, there is no soil disturbance, and the pressure of the machinery results in a more compacted surface layer when compared to systems that have soil disturbance [2]. However, root development was not affected, considering that there was no statistical difference between CT and NT in the production of RDB (Table 1).

As NT prioritizes the non-disturbing of the soil, as well as the permanent cover of the soil and crop rotation, it is probable that the radicular exploration of the different crops has favored the formation of biopores. The non-revolving may also have contributed to the greater continuity of the pores [27,28,29], thus not affecting the root development of soybean in depth, despite the high value of PR and Bd in NT. Since there is no impediment to the root system, the plant is able to explore a larger area of the soil, resulting in greater absorption of water and nutrients and, consequently, producing more.

The higher water retention capacity in the layer from 0.20 to 0.40 m in NT may mean a greater ability of plants to tolerate water stress, given that at this depth, NT has lower PR and Bd, as well as a higher amount of root than CT (Table 1), thus having easier access to water stored at depth, and consequently, suffering less stress in summer periods [30].

Despite CT having presented higher Mp (Table 2), both managements presented values below 0.10 m^3^ m^−3^, which is the minimum macroporosity value suitable for gas and liquid exchange between the external environment and the soil, considered critical for the development of root formation of most plant species [31]. Macropores are responsible for aeration, water movement in the soil profile, and also for root penetration [6]. Yet, despite NT presenting lower Mp, this did not influence soybean root development since it presented the highest amount of root dry biomass (Table 1).

In clay-textured soils, it is common to find reduced macroporosity due to the smaller specific surface of the particles [32,33]. The probability of macropore volume reaching critical values increases in clay-textured soils conducted in NT due to the absence of soil mobilization and by the accommodation of particles either naturally or by forces exerted on the soil surface [34,35].

It should be noted that, despite CT having presented higher Mp, the effect of mobilization on macroporosity does not persist for a long time. Most of the time, it is less than a year due to the reconsolidation that occurs in soils that have been mobilized and that receive successive cycles of wetting and drying [15,16,32].

Macroporosity is directly related to the process of water infiltration into the soil [36], and the greater the macroporosity, the greater the water infiltration rate. However, in this research, in an already consolidated management system (33 years), the lowest macroporosity found in NT (Table 2) did not influence the water infiltration rate because despite having lower macroporosity than CT, NT had a higher accumulated infiltration rate, which is associated with the higher value of n in the equation (Figure 1), which also contributes to higher BIR.

These results are related to the better continuity of pores in NT since it is common in this system for the surface layer to present lower macroporosity but with more continuous pores, thus reflecting the infiltration of water into the soil [4,29].

Although both systems store water above the PWP in all DAR evaluated in the surface layer of the soil (0.00–0.10 m), the value average of PWP in NT was 12.76% lower. This difference may be an indication of the possible vulnerability of NT in periods of prolonged summers. However, the storage capacity of NT was 21.43% greater than CT (Figure 2), thus being able to supply the need for water by the plants in periods of drought since it can store and make a greater amount of water available to the plants.

The non-availability of water at a depth of 0.20–0.40 m in NT (Figure 2) did not affect plant development, as 94.68% of the roots were between 0.00 and 0.20 m deep in the soil (Table 1), and this system showed higher soybean grain yield (Figure 4).

The low moisture in NT in this layer can be explained by the fact that the volume of rain incident in the two systems is the same, but in NT, there is greater retention by straw and soil organic matter (SOM) on the surface, since the FC in NT, in this layer, is higher (Figure 2). So much of the rain volume does not infiltrate the profile [37].

The results of water stored in the soil indicate that, in short periods of drought (53–65 and 117–132 days after sowing) (Figure 3), NT provides greater water supply to the surface roots, which agrees with the results found by [38] and [8]. In addition, better soil cover provided by NT can reduce water loss to the atmosphere via evaporation and maximize water use by plants [39,40], especially in the early cultivation stages, which would favor its storage in the soil. It is worth mentioning that this first dry period (53–65 days after sowing) was during the flowering and grain formation period, which is the period of greatest water demand for soybeans [41]. A greater amount of water in the soil, in layers with a higher nutrient concentration, increases the chances of better plant nutrition, contributing to greater productivity [42].

Thus, the highest soybean grain yield verified in NT (Figure 4) is associated with the values found in the physical evaluations of the soil, root distribution, and soil water dynamics since this soil management system presented superior results in relation to CT for these variables (Table 1, Figure 1, Figure 2 and Figure 3).

### 3.2. Correlation between Soil Physical and Hydraulic Properties, Soil Water, and Soybean Yield

The reduction in macroporosity and increase in microporosity are linked to soil compaction, hence the positive and negative correlations, respectively, with soybean grain yield in the 0.10–0.20 m layer (Table 3). Furthermore, soybean, perhaps more than other crops, is very dependent on macroporosity due to its need for aeration for maximum nodulation and biological N fixation, to which it is very dependent [43,44].

The higher macroporosity may have promoted better water infiltration into the soil, which was retained in the mesopores and thus is more easily available to plants due to the lower retention pressure when compared to micropores [45]. Thus, it is possible that in NT, there is a greater water absorption ease by plants, providing a more favorable water status and delaying the onset of water deficit in drought conditions.

The greater amount of root can reduce the MWC because there is greater absorption of (demand for) water, with greater loss by evapotranspiration. Thus, this explains the negative correlation of RDM with MWC at intermediate depth. On the other hand, the positive correlation of RDM with MWC and WI (Table 3) may indicate the formation of biopores in depth, facilitating water infiltration and the ability of the soil to store it.

If there is greater soil mechanical resistance to root penetration, it is expected that this soil has less pore space (Tp and Mp), resulting in lower water storage capacity (MWC), justifying the correlation found between PR and Tp, Mp, and MWC (Table 3). However, the positive correlation of the PR with WI indicates that, despite the soil having the MWC reduced by the compaction, this did not influence the water infiltration capacity in the soil in a long-term management system, but the infiltrated water was not available for the plants.

Tp is the sum of Mp and mp of the soil. So, by increasing Tp, it is expected that at least one of these properties (Mp and/or mp) will also increase. Increasing these characteristics is also expected to increase MWC since Mp is related to water infiltration and mp to soil retention. The reduction in Mp is indicative of compaction since it increases the density of the soil.

The negative correlation of Mp with WI and WSS in the surface layer of the soil (Table 3) may be related to the quality and continuity of the pores and not to the quantity since it is a long-term management system (33 years).

The negative correlation of Mp with mp at a depth of 0.10–0.20 m was expected since, if macroporosity increases, it is expected that microporosity and, therefore, WSS will decrease once the water stored in Mp is retained with lower retention force, making it more susceptible to loss [45]. The increase in microporosity is indicative of increased soil compaction, which may be the factor in the reduction in soybean grain yield in CT, as this factor directly affects crop production [18,22,46].

A soil with high density has little pore space, and this directly influences the ability of this soil to store water. However, it was expected that higher density would result in lower WI, which was not observed in this work. This way, as the soil was more compacted, consequently with less pore space, it may be that despite the water being able to infiltrate the first 0.20 cm of the soil, it was not possible to store it.

## 4. Materials and Methods

### 4.1. Location and History of the Experimental Area

The experiment was located in Botucatu, São Paulo State, southeastern Brazil (48°23′ W, 22°51′ S, 740 m asl), on a clay-textured Typic Rhodudalf soil [47]. The main soil chemical [48] and textural [49] properties are shown in Table 4.

**Figure 5:**
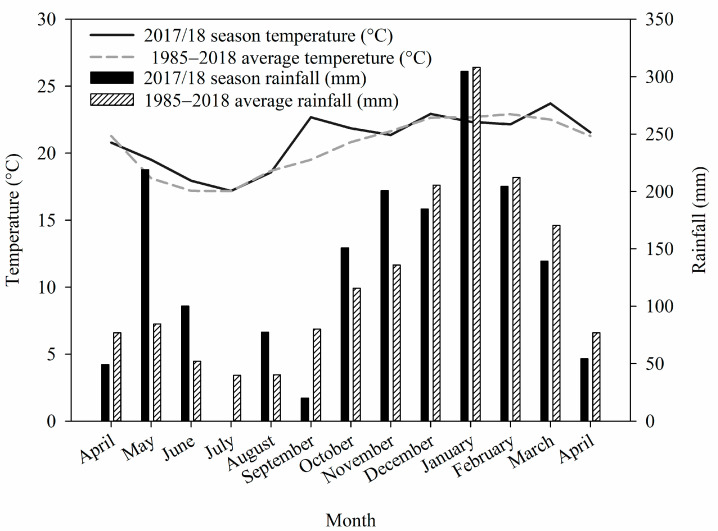
Monthly averages of temperature (°C) and rainfall (mm) between the years of 1985 and 2018 and during the 2017/18 harvest seasons.

The climate, according to the Köppen classification, is Cwa type (tropical, with a dry winter and a hot, rainy summer). The long-term (1985–2018) and 2017/18 growing season averages of monthly temperature and rainfall are shown in Figure 5. The data were obtained using an automatic weather station.

The experiment is part of a long-term study of the CT (disk plow plus disk harrow) and NT systems begun in the 1985/1986 growing season, and the management history is shown in Table 5.

### 4.2. Experimental Design and Conduction of the Experiment

The experimental design used was randomized complete blocks with four replications. The plots (50 m × 6.5 m) were constituted by the two soil management systems (CT and NT), and the subplots were composed by the three evaluated soil depths (0.00–0.10; 0.10–0.20; and 0.20–0.40 m).

The soybean cultivar TMG 7062 IPRO was mechanically sown on 8 December 2017, with a spacing of 0.45 m between rows and a density of 15 seeds m^−1^. The seeds were treated with carboxin + thiran fungicide, tiamethoxam insecticide, *Bradyrhizobium* sp. inoculant, and Co and Mo micronutrients. Doses of fungicide, insecticide, and micronutrients were defined based on the manufacturer’s recommendation. For seed inoculation, liquid inoculant was used at a dosage calculated to provide 1.2 million viable cells per seed. Sowing fertilization was conducted with 60 kg ha^−1^ of K_2_O and 60 kg ha^−1^ of P_2_O_5_, using KCl and single superphosphate as sources, respectively. In both soil management systems, sowing was carried out on the straw of the autumn–winter crop (black oats). In CT, soil tillage was carried out only in April before sowing black oats with a harrow and a leveler at a depth of 0.00–0.20 m (Table 5).

The phytosanitary management of soybeans involved weed control with the application of the herbicide glyphosate (1.8 kg a.i. ha^−1^) associated with the herbicide sethoxidim (1.25 kg a.i. ha^−1^). The fungicides pyraclostrobin + epoxiconazole (0.08 + 0.03 kg a.i. ha^−1^, respectively) and azoxystrobin + cyproconazole (0.06 + 0.024 kg a.i. ha^−1^, respectively) and the insecticides thiamethoxam + lambda-cialotrin (0.028 + 0.21 kg a.i. ha^−1^) were applied preventively. Pre-harvest plant killing was performed using the herbicide paraquat (0.4 kg a.i. ha^−1^) when the soybean was at the R7.3 phenological stage (when most of the seeds had a yellowish coat with a shiny surface and were already detached from the pod).

The soybean grain yield was estimated at 111 days after sowing when plants were harvested from 4.5 m^2^ of the useful area of each subplot. The material was threshed on stationary equipment, and the grains were weighed to calculate yield, correcting the humidity to 130 g kg^−1^.

### 4.3. Sampling of Soybean Roots

The soybean plant roots were collected at the R2 phenological stage (full flowering). Sampling was carried out by collecting soil containing roots using a cylindrical auger with a diameter of 50 mm. Collections were carried out at depths of 0.00–0.10; 0.10–0.20; and 0.20–0.40 m. In each subplot, the samples from each depth were composed of four subsamples, and were collected between the rows at a distance of approximately 0.10 m from the seeding line. After collection, the portions of soil containing the roots were poured into sieves with a mesh size of 1 mm and washed with a jet of water. The roots retained on the sieves were collected with tweezers, placed in paper bags, and dried in a forced aeration oven at 60 °C for 48 h to determine the dry biomass (kg ha^−1^).

### 4.4. Soil Assessments

The WC was determined between 3 January 2018 and 18 April 2018 using a capacitance probe (Diviner^®^ model, Sentek Pt Ltd., Stepney, SA, Australia) inserted into access tubes previously installed in the areas. One tube was installed in the center of each plot. WC monitoring was carried out in the 0–0.10, 0.10–0.20, and 0.20–0.40 m depth layers, with readings always happening after rainfall, which were repeated at one, three, five, eight, and fifteen days after the occurrence of rain (DAR). Only rains above 10 mm were considered.

With the average values of soil moisture in each reading period, it was possible to determine the WC stored at each soil depth. The available water for plant uptake was considered as that which was located between the FC and PWP. Eighteen readings were performed with 1 DAR, five readings with 3 DAR, four readings with 5 DAR, four readings with 8 DAR, and three readings with 15 DAR. The monitoring of the volume of rain was carried out via observations in a rain gauge installed in the experimental area [50].

At the time of root collections, soil penetration resistance tests (PR) were carried out at three points per subplot using the Impact Penetrometer (model IAA/Planalsucar–Stolf, Piracicaba, SP, Brasil) [50].

The Bd, Mp, mp, Tp, FC, and PWP of the soil were determined in samples with undeformed structures collected in duplicate for each subplot [49,51,52]. For this, trenches that were 0.50 m deep by 0.50 m wide and 0.50 m long were opened to collect the samples using volumetric rings that were 0.05 m high and 0.048 m in diameter.

Soil moisture in FC and PWP were determined using a Richards chamber, considering the matrix potentials of −0.03 and −1.5 MPa, respectively. With the FC and PWP values, it was possible to calculate the MWC by subtracting the PWP moisture from the FC moisture value [53].

WI was evaluated by the concentric ring infiltrometer method, according to [54]. This methodology is based on the use of two rings of 0.30 m in height, one with 0.30 m and the other with 0.60 m in diameter, positioned concentrically on the ground. The rings were driven vertically 0.15 m into the ground. After filling the total volume of the rings with water, the infiltration reading was performed on the inner ring with the aid of a ruler. The reading was performed until the infiltration rate in the inner ring became constant, that is, with five equal measurements with an interval of 30 min. Infiltration readings were taken at the following time intervals: five readings at one-minute intervals; five readings at two-minute intervals; five readings at five-minute intervals; five readings at ten-minute intervals; five readings at fifteen-minute intervals; five readings at twenty-minute intervals; and finally, five readings with intervals of thirty minutes, to confirm stabilization in the infiltration rate, determining the WI accumulated over time, based on Equation (1) [55,56].

WI = k × T^n^ (1)

where WI is the infiltrated depth in time (cm); T is the time (min); k is the soil-dependent constant; and n is the soil-dependent constant ranging from 0 to 1.

The water infiltration readings were adjusted by the infiltration equations according to the mathematical models proposed by Kostiakov–Lewis [55,56]. Equation (2) was used to obtain the BIR as follows:

BIR = 60 × k × n × T^n-t^ (2)

where BIR is the infiltration rate of water in the soil (cm h^−1^); T is the time (min); k is the soil-dependent constant; and n is the soil-dependent constant ranging from 0 to 1.

To determine the coefficients and exponents of the potential equations, the linear regression method was used, applying the logarithms on both sides of the potential equation, resulting in Equation (3):

Log I = log a + log t(3)

Equation (3) was transformed into a linear Equation (4) as shown below:

I = A + Bn(4)

where n was defined by Equation (5).

n = {∑xy − [(∑x × ∑y)/N]}/{∑x^2^ − [(∑x)^2^/N]} (5)

where n is the number of readings performed.

Thus, Equation (6) was defined as follows:

A = Y − X(6)

### 4.5. Statistical Analysis

Data were tested for normality using the Anderson–Darling test and for homoscedasticity using the Levene test. Subsequently, the data were submitted for analysis of variance and *t*-test for means comparison (*p* < 0.05). For the variables water infiltration, water stored in the soil, and soybean grain yield were not considered in subplots since they are not dependent on soil depth.

The data were also subjected to Pearson’s correlation analysis. WC data were analyzed using the nonparametric Mann–Whitney test (*p* < 0.01). Statistical analyses were performed using the R software (version 13.0).

## 5. Conclusions

The physical properties of the soil were influenced by management systems, interfering with soil water dynamics, root growth, and grain yield of soybean crop. Surface soil compaction in the no-tillage system did not impair root development and grain yield of soybean crops. Since the long-term no-tillage system (33 years) offers less risk of water stress to soybean plants, it contributes to greater grain yield of this crop when compared to the conventional tillage system.

Our study proves that changes in the root growth environment promoted by soil management systems are determinants of soybean yield. Future research that better addresses the architecture and continuity of pores provided by no-tillage systems and evaluations of soybean plant development (physiological, biochemical, and even molecular parameters) may contribute to a better understanding of soil–plant relationships in long-term no-tillage.

## Figures and Tables

**Table 1:** Average values of penetration resistance, bulk density, maximum available water capacity in the soil and soybean root dry biomass (RDB) at depths of 0.00–0.10, 0.10–0.20, and 0.20–0.40 m in an experiment of long-term management systems.

Soil Management	Soi Depth (m)		
	0.00–0.10	0.10–0.20	0.20–0.40
	Soil penetration resistance (MPa)		
Conventional tillage	0.71 ± 0.48 bB	1.62 ± 0.57 bB	3.52 ± 1.30 aA
No-tillage	4.11 ± 0.69 aAB	4.40 ± 0.70 aA	3.33 ± 0.73 aB
	Soil bulk density (g cm^−3^)		
Conventional tillage	1.04 ± 0.09 bB	1.27 ± 0.04 aA	1.24 ± 0.03 aA
No-tillage	1.30 ± 0.05 aAB	1.35 ± 0.03 aA	1.26 ± 0,07 aB
	Maximum available water capacity (cm^3^ cm^−3^)		
Conventional tillage	0.054 ± 0.08 aB	0.069 ± 0.05 aA	0.047 ± 0.08 bC
No-tillage	0.047 ± 0.06 bB	0.037 ± 0.03 bC	0.066 ± 0.06 aA
	Soybean root dry biomass (kg ha^−1^)		
Conventional tillage	1110 ± 48.44 aA	136 ± 13.62 bB	49 ± 11.47 aB
No-tillage	1316 ± 57.19 aA	1009 ± 94.00 aA	13 ± 16.39 aB

Means followed by a lowercase letter in the column and a capital letter in the row do not differ from each other by Student’s *t*-test (*p* < 0.05).

**Table 2:** Values of macroporosity, microporosity, and total porosity of the soil at depths of 0.00–0.10, 0.10–0.20, and 0.20–0.40 m in an experiment of long-term management systems.

Treatment	Macroporosity	Microporosity	Total Porosity
	cm^3^ cm^−3^		
Soil management			
Conventional tillage	0.09 ± 0.02 a	0.44 ± 0.02 a	0.53 ± 0.02 a
No-tillage	0.06 ± 0.02 b	0.44 ± 0.02 a	0.50 ± 0.02 a
Depth (m)			
0.00–0.10	0.09 ± 0.02 a	0.43 ± 0.02 a	0.52 ± 0.01 a
0.10–0.20	0.07 ± 0.02 a	0.44 ± 0.02 a	0.51 ± 0.01 a
0.20–0.40	0.07 ± 0.02 a	0.44 ± 0.02 a	0.51 ± 0.01 a

Means followed by a lowercase letter in the column do not differ by Student’s *t*-test (*p* < 0.05).

**Table 3:** Correlation between root dry biomass (RDB), soil penetration resistance (PR), total porosity (Tp), macroporosity (Mp), microporosity (mp), bulk density (Bd), maximum available water capacity (MWC), soybean grain yield (SY), accumulated water infiltration (WI), and water stored in the soil (WSS) in conventional tillage and long-term no-tillage systems at depths of 0.00–0.0–10, 0.10–0.20, and 0.20–0.40 m.

Variables	RDB	PR	TP	Mp	mp	Bd	MWC	SY	IA
	0.00–0.10 m								
PR	0.431 ^ns^								
Tp	−0.195 ^ns^	−0.786 *							
Mp	0.013 ^ns^	−0.828 *	0.899 **						
mp	−0.456 ^ns^	0.143 ^ns^	0.166 ^ns^	−0.283 ^ns^					
Bd	0.077 ^ns^	0.704 ^ns^	−0.670 ^ns^	−0.741 *	0.201 ^ns^				
MWC	−0.333 ^ns^	−0.905 **	0.804 *	0.882 **	−0.255 ^ns^	−0.835 *			
SY	−0.224 ^ns^	−0.116 ^ns^	−0.161 ^ns^	−0.095 ^ns^	−0.138 ^ns^	−0.348 ^ns^	0.208 ^ns^		
WI	0.304 ^ns^	0.850 **	−0.689 ^ns^	−0.785 *	0.260 ^ns^	0.785 *	−0.938 **	−0.475 ^ns^	
WSS	−0.349 ^ns^	0.483 ^ns^	0.605 ^ns^	−0.807 *	0.493 ^ns^	0.437 ^ns^	−0.562 ^ns^	−0.006 ^ns^	0.588 ^ns^
	0.10–0.20 m								
PR	0.847^**^								
Tp	−0.015 ^ns^	0.231 ^ns^							
Mp	−0.104 ^ns^	−0.155 ^ns^	0.236 ^ns^						
mp	0.082 ^ns^	−0.025 ^ns^	0.497 ^ns^	−0.725 *					
Bd	0.451 ^ns^	0.601 ^ns^	−0.527 ^ns^	−0.692 ^ns^	0.244 ^ns^				
MWC	−0.932 **	−0.967 **	0.159 ^ns^	0.158 ^ns^	−0.029 ^ns^	−0.632 ^ns^			
SY	−0.291 ^ns^	−0.143 ^ns^	−0.087 ^ns^	0.781 *	−0.758 *	−0.447 ^ns^	0.208 ^ns^		
WI	0.941 **	0.888 **	−0.158 ^ns^	0.349 ^ns^	0.199 ^ns^	0.644 ^ns^	−0.938 **	−0.475 ^ns^	
WSS	−0.250 ^ns^	−0.565 ^ns^	−0.091 ^ns^	−0.189 ^ns^	0.104 ^ns^	−0.079 ^ns^	0.434 ^ns^	−0.066 ^ns^	−0.260 ^ns^
	0.20–0.40 m								
PR	−0.641 ^ns^								
Tp	−0.366 ^ns^	0.515 ^ns^							
Mp	0.463 ^ns^	0.444 ^ns^	0.818 *						
mp	0.100 ^ns^	0.372 ^ns^	0.771 *	0.264 ^ns^					
Bd	0.164 ^ns^	0.326 ^ns^	0.097 ^ns^	−0.358 ^ns^	0.559 ^ns^				
MWC	0.893 **	−0.299 ^ns^	−0.199 ^ns^	−0.429 ^ns^	0.141 ^ns^	0.169 ^ns^			
SY	−0.104 ^ns^	−0.268 ^ns^	−0.161 ^ns^	−0.069 ^ns^	−0.193 ^ns^	0.197 ^ns^	−0.208 ^ns^		
WI	0.875 **	−0.271 ^ns^	−0.115 ^ns^	−0.291 ^ns^	0.129 ^ns^	−0.070 ^ns^	0.939 **	−0.475 ^ns^	
WSS	0.516 ^ns^	0.224 ^ns^	0.563 ^ns^	0.886 **	−0.036 ^ns^	−0.586 ^ns^	−0.638 ^ns^	−0.096 ^ns^	−0.455 ^ns^

ns not significant, ** significant at 1%, and * significant at 5% via Pearson’s correlation analysis.

**Table 4:** Chemical and granulometric analysis of the soil managed under conventional tillage and long-term no-tillage system at depth 0.00–0.20 m.

Management System	pH(CaCl_2_)	P_resin_	S	H + Al	Ca	Mg	K	Sand	Silt	Clay
		mg dm^−3^		mmol_c_ dm^−3^				g kg^−1^		
Conventional tillage	5.0	61.2	3.6	36.3	39.5	12.7	4.7	147	239	614
No-tillage	5.4	84.4	4.4	29.6	43.5	14.8	3.3			

pH(CaCl_2_): active acidity in an 0.01 M calcium chloride solution; P: exchangeable phosphorus; H + Al: potential acidity; Ca: exchangeable calcium; Mg: exchangeable magnesium; K: exchangeable potassium mg dm^−3^: milligram per cubic decimeter; mmol_c_ dm^−3^: millimol charge per cubic decimeter; g kg^−1^: gram per kilogram.

**Table 5:** Soil management system and crop rotation.

Year	Management System				Crop Sequence Fall–Winter/Spring–Summer
	Conventional Tillage		No-Tillage		
	Fall	Spring	Fall	Spring	
1985/86	Plowing + harrowing	Plowing + harrowing	Plowing + harrowing	No-tillage	Wheat/soybean
1986/87 to 1994/95	Plowing + harrowing	Plowing + harrowing	No-tillage	No-tillage	Wheat/soybean
1995/96 to 1998/99	Without soil mobilization	Without soil mobilization	No-tillage	No-tillage	Fallow/fallow
1999/00	Plowing + harrowing	Plowing + harrowing	No-tillage	No-tillage	Black oat/maize
2000/01 and 2001/02	Without soil mobilization	Without soil mobilization	No-tillage	No-tillage	Fallow/fallow
2002/03 and 2003/04	Plowing + harrowing	Plowing + harrowing	No-tillage	No-tillage	Black oat/millet-bean
2004/05 and 2005/06	Plowing + harrowing	Plowing + harrowing	No-tillage	No-tillage	Black oat/maize
2006/07	Without soil mobilization	Without soil mobilization	No-tillage	No-tillage	Fallow/soybean
2007/08	Plowing + harrowing	Without soil mobilization	No-tillage	No-tillage	Yellow oat/bean
2008/09	Plowing + harrowing	Without soil mobilization	No-tillage	No-tillage	Yellow oat/bean
2009/10 to 2011/12	Plowing + harrowing	Without soil mobilization	No-tillage	No-tillage	Black oat/maize + brachiaria
2012/13	Without soil mobilization	Plowing + harrowing	No-tillage	No-tillage	Brachiaria/soybean
2013/14	Without soil mobilization	Plowing + harrowing	No-tillage	No-tillage	Wheat/soybean
2014/15	Without soil mobilization	Plowing + harrowing	No-tillage	No-tillage	Safflower/soybean
2015/16	Without soil mobilization	Plowing + harrowing	No-tillage	No-tillage	Safflower/maize
2016/17	Plowing + harrowing	Without soil mobilization	No-tillage	No-tillage	Black oat/maize
2017/18	Plowing + harrowing	Without soil mobilization	No-tillage	No-tillage	Black oat/soybean

## Data Availability

Not applicable.
